# Evaluation of West Nile Virus Diagnostic Capacities in Veterinary Laboratories of the Mediterranean and Black Sea Regions

**DOI:** 10.3390/pathogens9121038

**Published:** 2020-12-11

**Authors:** Elisa Pérez-Ramírez, Cristina Cano-Gómez, Francisco Llorente, Ani Vodica, Ljubiša Veljović, Natela Toklikishvilli, Kurtesh Sherifi, Soufien Sghaier, Amel Omani, Aida Kustura, Kiril Krstevski, Ilke Karayel-Hacioglu, Naglaa Mohamed Hagag, Jeanne El Hage, Hasmik Davdyan, Mohd Saddam Bintarif, Bojan Adzic, Nabil Abouchoaib, Miguel Ángel Jiménez-Clavero, Jovita Fernández-Pinero

**Affiliations:** 1 Centro de Investigación en Sanidad Animal, Instituto Nacional de Investigación y Tecnología Agraria y Alimentaria (INIA-CISA), 28130 Valdeolmos, Spain; cristina.cano@inia.es (C.C.-G.); dgracia@inia.es (F.L.); majimenez@inia.es (M.Á.J.-C.); fpinero@inia.es (J.F.-P.); 2 Department of Animal Health, Food Safety and Veterinary Institute, Tirana, Albania; anivodica@hotmail.com; 3 Virology Department, Scientific Institute of Veterinary Medicine of Serbia, 11000 Belgrade, Serbia; ljub.veljovic@gmail.com; 4 Laboratory of Virology and Molecular Biology, LEPL State Laboratory of Agriculture (SLA), 0159 Tbilisi, Georgia; Natela.Toklikishvili@sla.gov.ge; 5 Department of Veterinary Medicine, Faculty of Agriculture and Veterinary Sciences, University of Prishtina “Hasan Pristhina”, 10000 Prishtine, Kosovo; kurtesh.sherifi@uni-pr.edu; 6 Virology Department, Institute of Veterinary Research of Tunisia, 1006 Tunis, Tunisia; sghaiersoufien@yahoo.fr; 7 Laboratoire Central Vétérinaire d’Alger, Institut National de la Médecine Vétérinaire, Algiers, Algeria; omani_amel@yahoo.fr; 8 Veterinary Faculty, University of Sarajevo, 71000 Sarajevo, Bosnia and Herzegovina; a.kustura@vfs.unsa.ba; 9 Faculty of Veterinary Medicine, Ss. Cyril and Methodius University, 1000 Skopje, North Macedonia; krstevski@fvm.ukim.edu.mk; 10 Virology Department, Faculty of Veterinary Medicine, Ankara University, 06110 Ankara, Turkey; ilkeekarayel@gmail.com; 11 Animal Health Research Institute, Dokki 12618, Egypt; NaglaaHagagAHRI@gmail.com; 12 Animal Health Laboratory, Lebanese Agricultural Research Institute, 90-1064 Fanar, Lebanon; jeannehage@gmail.com; 13 Republican Veterinary-Sanitary and Phytosanitary Center of Laboratory Services SNCO, Yerevan, Armenia; hasmik58.vetlab@yandex.ru; 14 Animal Wealth Laboratory Sector, Ministry of Agriculture, Amman, Jordan; mbintarif@yahoo.com; 15 Diagnostic Veterinary Laboratory, 81000 Podgorica, Montenegro; bojan.adzic@vetlab.co.me; 16 Casablanca Regional Research and Analysis Laboratory of National Office of Sanitary Safety and Food Products (ONSSA), Nouaceur, 20 000 Casablanca, Morocco; nabilabouchoaib@gmail.com; 17 CIBER Epidemiología y Salud Pública (CIBERESP), 28029 Madrid, Spain

**Keywords:** West Nile virus (WNV), Usutu virus (USUV), flavivirus, external quality assessment (EQA), MediLabSecure, diagnostics, PCR, ELISA

## Abstract

The increasing incidence of West Nile virus (WNV) in the Euro-Mediterranean area warrants the implementation of effective surveillance programs in animals. A crucial step in the fight against the disease is the evaluation of the capacity of the veterinary labs to accurately detect the infection in animal populations. In this context, the animal virology network of the MediLabSecure project organized an external quality assessment (EQA) to evaluate the WNV molecular and serological diagnostic capacities of beneficiary veterinary labs. Laboratories from 17 Mediterranean and Black Sea countries participated. The results of the triplex real time RT-PCR for simultaneous detection and differentiation of WNV lineage 1 (L1), lineage 2 (L2) and Usutu virus (USUV) were highly satisfactory, especially for L1 and L2, with detection rates of 97.9% and 100%, respectively. For USUV, 75% of the labs reported correct results. More limitations were observed for the generic detection of flaviviruses using conventional reverse-transcription polymerase chain reaction (RT-PCR), since only 46.1% reported correct results in the whole panel. As regards the serological panel, the results were excellent for the generic detection of WNV antibodies. More variability was observed for the specific detection of IgM antibodies with a higher percentage of incorrect results mainly in samples with low titers. This EQA provides a good overview of the WNV (and USUV) diagnostic performance of the involved veterinary labs and demonstrates that the implemented training program was successful in upgrading their diagnostic capacities.

## 1. Introduction

West Nile virus (WNV) is an enveloped spherical, single-stranded positive-sense RNA virus belonging to the *Flaviviridae* family [1]. It is maintained in nature in an enzootic cycle involving ornithophilic mosquitoes (mainly *Culex*) as transmission vectors and certain birds as reservoir hosts. Spill-over from this cycle occasionally results in severe outbreaks in horses and humans that are considered dead-end hosts, which means that they cannot transmit the virus to feeding mosquitoes due to their low and transient viremia [2].

Although most human infections are asymptomatic, in some instances (~20%) the virus can cause a febrile syndrome (WNV fever). Around 1% of the cases progress to severe neuro-invasive disease with a fatality rate of around 10% [3]. In horses, the infection is generally asymptomatic, but approximately 10% of the infected animals develop neurological symptoms such as ataxia, limb paralysis, skin fasciculation and muscle tremors [4]. In birds, the pathogenic potential greatly differs among species and also depends on the viral strain. Passerine birds (especially corvids) and some raptor species are particularly susceptible to WNV infection [5].

Phylogenetic studies have revealed the existence of at least seven genetic lineages, of which lineages 1 (L1) and 2 (L2) are the most widespread and relevant for human and animal health [6]. Circulation of the virus has been regularly reported in wide areas of the Euro-Mediterranean area since 1998 [7]. However, in recent years, the virus has dramatically expanded, with an upsurge in the number and incidence of outbreaks in humans and animals caused by both L1 and L2 strains. In fact, in 2018 the transmission season started earlier and the number of human autochthonous cases reported in EU and neighboring countries (2083 cases) exceeded the global number from the previous seven years. In horses, 285 cases were notified, which represents an increase of 30% in comparison with 2017 [8]. However, epidemiological data on WNV from Black Sea countries is scarce and therefore surveillance efforts in this region needs to be harmonized with those implemented in other countries.

The epidemiological situation in the Mediterranean basin is complex because, apart from WNV L1 and L2, other flaviviruses circulate in overlapping areas [6,9]. This is the case with Usutu virus (USUV), a zoonotic arbovirus closely related to WNV that has spread throughout Europe since 2001, when it was first detected in Austria [10]. Nowadays many countries have reported the presence of both viruses in mosquitos, birds and humans [9,11]. Of the countries participating in this EQA, in at least three (Serbia, Tunisia and Morocco) the co-circulation of both viruses has been demonstrated [12,13,14].

WNV diagnostic methods include virus isolation, reverse-transcription polymerase chain reaction (RT-PCR) and serological tests. Isolation procedures are laborious and require biosecurity level 3 (BSL-3) facilities. By contrast, molecular methods, such as RT-PCR, and particularly real-time RT-PCR (RRT-PCR), can be easily applied in basic laboratories, are fast and sensitive, enabling timely detection and early outbreak response [6]. In the current context, RRT-PCRs that allow for simultaneous detection of WNV L1, L2 and other flaviviruses such as USUV are extremely useful for outbreak investigations and epidemiological studies, maximizing the information obtained from each sample [15].

Among the antibody detection tools, ELISA tests are the most widely used with several commercial kits available. However, a relevant limitation of WNV ELISA tests is the cross-reactivity of antibodies raised against different flaviviruses that can lead to diagnostic misinterpretations [6,16]. To confirm WNV infection, the gold standard method is virus neutralization (VNT) that enables differential diagnosis by titration of neutralizing antibodies in parallel against different flaviviruses that could cross-react in serological tests. Nevertheless, VNT is time-consuming and has to be performed in BSL-3 labs.

Prevention and control efforts substantially rely on effective surveillance of the infection in animals and vectors that can act as early warning triggers [17]. The implementation of locally adapted surveillance systems in birds, horses and mosquitos and the upgrade of the diagnostic capacities of veterinary laboratories is crucial to fight the disease.

MediLabSecure is an EU-funded project whose main objective is to create a framework for collaboration to promote arbovirus surveillance under a One Health approach in 19 countries of the Mediterranean and Black Sea regions [18,19]. Since the beginning of the project in 2014, the MediLabSecure animal virology network has implemented numerous actions to enhance capacity building of veterinary labs to face health threats caused by emerging arboviruses.

In a specific questionnaire delivered to identify the priorities of the beneficiary countries in the field of arboviral diseases, all the labs recognized WNV as a common health priority in the region. A training curriculum was implemented to improve diagnostic performance of the veterinary labs for this pathogen, including two diagnostic workshops (molecular and serological diagnosis) that were organized in 2015 and 2016 at Centro de Investigación en Sanidad Animal (INIA-CISA) (Madrid, Spain). After these training sessions, an external quality assessment (EQA) was organized between October 2016 and March 2017 to evaluate the degree of learning and the capacity of the labs to incorporate the molecular and serological techniques into their routine diagnostic activities. In this study we report the results of this inter-laboratory trial and provide relevant information about the current WNV (and USUV) diagnostic capacities of veterinary labs in the Mediterranean and Black Sea regions. This exercise also enabled an extensive reproducibility assessment of the recommended tests for WNV and USUV diagnostics.

## 2. Results

**Figure 1:**
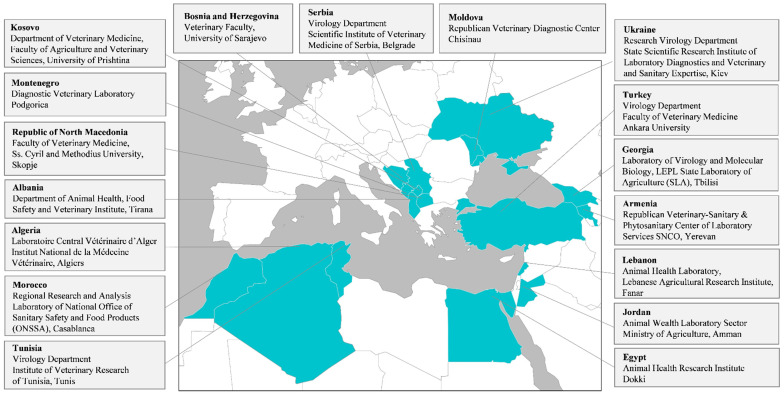
Participant laboratories. Obtained from [20].

Seventeen laboratories submitted results, representing 17 countries from the Mediterranean and Black Sea regions (Figure 1).

### 2.1. Virus Genome Detection

A total of 13 datasets were received from 13 labs (76.4% of response) for the generic detection of flaviviruses. The results of all labs are shown in Table 1. Four labs did not carry out this technique due to lack of specific equipment to perform conventional RT-PCR assays and were therefore unable to detect the flavivirus positive sample of the panel corresponding to Japanese encephalitis virus (JEV).

Out of the 13 datasets, only 6 (46.1%) were 100% correct, i.e., identified as positive the eight samples containing WNV L1, L2, USUV or JEV and as negative the two negative samples of the panel. However, it should be noted that for three of the positive samples, weak positive results (weak bands) were expected. The two samples (W2 and W8) with expected strong positive bands were correctly identified by all the labs (Table 1). Three false positive results were reported by two labs.

For the specific detection of WNV and USUV by RRT-PCR, we received 18 datasets from the 17 participating labs (100% of response), including one double dataset from lab #16 that used an alternative method [21] apart from the recommended one. The results of the labs using the recommended method are shown in Table 2. Overall, the results of the triplex RRT-PCR were very good, with the exception of one laboratory (#1) that reported incorrect results in the whole panel (Table 2). If we exclude the results of this lab from the global analysis of the EQA, out of 16 labs, 12 (75%) reported 100% concordant results, which means that they correctly identified WNV L1, L2 and USUV in all the positive samples (including co-infections).

However, a technical limitation was observed in the application of the triplex RRT-PCR in two labs (#8 and #12), because the Cy5 fluorescent channel necessary for the detection of USUV was lacking in the available real-time thermocyclers. Interestingly, one of these labs was able to overcome this limitation by applying an alternative conventional RT-PCR for the specific detection of USUV [22], obtaining 100% correct identification of USUV positive samples.

In general, highly satisfactory results were obtained for WNV L1 and L2 while more difficulties were observed for the correct identification of USUV positive samples (W3, W6 and W8) as shown in Table 2. In fact, out of the eight false negative results reported by four labs, seven corresponded to USUV and one to WNV L1.

Excluding results from lab #1, co-infections were successfully detected in 87.5% of cases for sample W3 (WNV L1+USUV) and in 81.2% of cases for sample W8 (WNV L2+USUV). The sample containing a related flavivirus (JEV) was correctly identified as negative by all the labs. Overall, the reported Ct values for the three viruses were in line with the reference values, except for labs #3 and #10 that reported lower Ct values for WNV L2 and labs #2 and #17 that reported higher Ct values than expected for WNV L2 and USUV in three samples. With regard to the negative samples, and excluding lab #1, only one laboratory reported a false positive result. Differences in qualitative results or Ct values were not attributable to a specific thermocycler.

Lab #16 correctly identified the WNV positive samples of the panel using a pair of alternative RT-PCR methods for specific detection of WNV L1 and L2 [21].

### 2.2. Antibody Detection

A total of 17 datasets were received from the 17 labs (100% response) for the generic detection of WNV antibodies using commercial competition ELISAs. Sixteen labs used the recommended method (INgezim West Nile Compac ELISA) and one lab (#8) applied an alternative commercial kit (ID Screen West Nile Competition Multi-species, IDvet).

All the labs that used the Ingenasa kit reported 100% concordant results, except for one negative sample that was assigned as doubtful by lab #7. The only lab that used the IDVet kit also reported 100% correct results.

For the specific detection of WNV IgM antibodies, 19 datasets were received from 17 labs (100% response). Sixteen labs used the recommended method (INgezim WNV IgM ELISA), and two (#14 and #16) also analyzed the panel with an alternative MAC ELISA, the ID Screen West Nile IgM Capture kit from IDvet. Laboratory #8 used this kit instead of the recommended one.

The results of the IgM antibody detection using the recommended method are presented in Table 3. Correct results in the whole panel were reported by 56.2% of the labs. It is important to note that, of the 4 IgM positive sera, two (W2 and W8) had low IgM antibody titers, which might explain the difficulties of some labs in identifying these samples. In fact, these sera displayed OD values close to the reference limits producing a higher percentage of false negative results (Table 3). The serum with high IgM antibody titer (W4) was successfully identified by all labs except one (#4). The six IgM negative sera were correctly assigned by all the labs except for one sample (W6) that was reported as IgM doubtful by lab #15.

As explained earlier, three labs used an alternative ELISA kit to analyze the panel. In all of them this kit failed to detect three out of the four IgM positive sera of the panel. Only the sample with high IgM antibody titer (W4) could be correctly identified by the IDVet IgM capture ELISA in the three labs.

## 3. Discussion

Despite the increasing incidence of WNV in Europe and the Mediterranean area, few external quality assessments have been organized to evaluate the diagnostic capacities of labs, especially in the veterinary sector. In the human health sector, five EQAs were organized by the ENIVD and EVD-LabNet networks between 2006 and 2017 to evaluate the WNV diagnostic performance of human virology labs in different countries, mostly in Europe but also in Middle East and America [23,24,25,26,27]. In Italy, two EQAs were organized in 2010 and 2011 by the National Institute of Health to assess the capacity of the national blood transfusion centers to detect WNV genome on blood donations [28]. In the veterinary sector, only two inter-laboratory assays were carried out in 2010 and 2013 to evaluate the capacity of the National Reference labs for equine diseases in Europe and Morocco to detect WNV antibodies in horse sera [29].

The EQA we present here has a number of differential features with respect to prior WNV inter-laboratory trials. In the first place, the geographical coverage of the involved laboratories. In previous EQAs, most of the participant labs were European while in this case labs from 17 non-EU countries including North Africa, Balkans, Black Sea and Middle East regions have been involved. Of these, only one lab in Bosnia and Herzegovina had participated in the previous serology EQA organized by ANSES in 2013 [29]. Secondly, this EQA was organized as a final evaluation after a three year training period where the participant labs attended several hands-on workshops aimed at improving their diagnostic performance. Thirdly, as the diagnostic assay used for molecular detection of WNV is a triplex RRT-PCR that allows the simultaneous detection of USUV, this EQA also provides relevant data about the capacities of the labs to identify this emerging flavivirus. Last, but not least, the labs were provided with all the materials required to perform the recommended diagnostic assays. The objective was to facilitate as much as possible the participation of all beneficiary labs. Moreover, and to promote sustainability, positive extraction and PCR controls were also provided to be used as quality controls during this EQA but also to serve as reference material for the future diagnostic activities of the labs. Such material is otherwise difficult to obtain and was greatly appreciated by the participants.

An added value of this EQA is also the combination of molecular and serological methods since this integrated approach is essential for WNV surveillance in animal populations. Although previous EQAs had analyzed the performance of vet labs to detect WNV antibodies, this is the first international proficiency test to evaluate the molecular diagnostic capacities of animal diagnostic labs.

The panel for WNV genome detection consisted of 10 samples containing various concentrations of four different flavivirus strains: two WNV European strains representing lineages 1 and 2, one European USUV strain and the reference JEV Nakayama strain. Unlike previous WNV EQAs where the viruses were diluted in human plasma [23,27,28] or virus culture medium [24], in this case the viruses were spiked in different organs’ homogenates, serum or blood from horses and birds to mimic as much as possible the clinical samples that the vet labs would analyze during real surveillance and outbreak investigations. Considering the current situation in many Mediterranean countries where different lineages of WNV co-circulate with other flaviviruses, two samples containing both WNV and USUV were included in the panel to evaluate the ability of the labs to identify coinfections.

The panel for antibody detection consisted of 10 sera from WNV infected horses with different IgG and IgM antibody titers. Overall, both panels conformed a very comprehensive proficiency test that allowed accurate evaluation of the WNV and USUV diagnostic capacities of the labs.

Some limitations were observed in the generic detection of flaviviruses. On the one hand, four laboratories (23.5%) did not perform this PCR. Apparently, in these labs real-time PCR has completely replaced conventional PCR and they even lack the necessary equipment to carry out the electrophoresis. This fact can have a negative impact in their diagnostic capacities for certain pathogens. In this case, they were unable to identify the presence of a non-WNV/USUV flavivirus in sample W9 (JEV). On the other hand, out of the 13 labs that performed the pan-flavivirus conventional RT-PCR, only 46.1% reported correct results in the whole panel, identifying the eight samples containing WNV, USUV or JEV. Most of the mistakes were false negative results, particularly in the three samples with lower viral load, for which weak positive bands were expected.

The performance of the labs with the triplex RRT-PCR was highly satisfactory in general terms, with the exception of lab #1 that reported incorrect results in all the samples. It seems that an error in the numbering of the tubes or during the transcription of the results could occur, as the reported result for each sample corresponded to the expected outcome of the preceding sample. Excluding this lab, the overall results for the specific detection of WNV L1 were excellent. The positive samples were identified by all labs except one false negative result in one lab, which represents an overall detection rate of 97.9%, much higher than that reported in previous EQAs organized by ENIVD and EVD LabNet networks in human virology labs [27,30]. With regard to the diagnosis of L2 WNV infections, these previous interlaboratory assays had evidenced important limitations in the participant labs. In the EQA organized in 2006 only 46.6% of the labs were able to detect L2 positive samples [23]. Although this percentage increased during the second ENIVD EQA in 2011, one third of the labs still failed to identify WNV L2 [24]. In the last EQA organized in 2017, the human labs had considerably improved the detection of WNV L2 but some false negative results were still reported [27]. In the present study, all the labs (except lab #1) successfully detected the presence of L2 in the positive samples of the panel. In the current epidemiological context, where L2 is already present in many Euro-Mediterranean countries and will probably expand to new territories [31,32], these results are highly relevant to ensure a timely detection of L2 circulation in animal populations of the involved countries.

As regards USUV detection, before this EQA, only two ring trials for human labs had included one USUV sample in the diagnostic panels [27,28]. Interestingly, in the EQA organized for blood transfusion centers in Italy in 2011, the USUV sample was misidentified as WNV positive by all the labs, indicating the presence of cross-reactivity in the two automated nucleic acid assays used [28]. Cross-reactions between both flaviviruses have been evidenced in several studies where automated commercial PCR kits were used to test human blood [33,34]. However, in the present EQA, USUV positive sample was reported as WNV negative by all the labs. This, together with the fact that the JEV sample was negative for the three viruses in all the labs, confirms the high specificity of the applied triplex PCR assay.

In the more recent EQA organized by EVD LabNet, the USUV sample was correctly detected by a small percentage of labs, even in countries with demonstrated USUV circulation, revealing a clear need for technical improvement in USUV diagnosis in participating labs [27]. In our EQA, 75% of the labs were able to identify the three positive samples and 81.2% detected two of them. Even so, two labs (11.7% of the total) could not detect the virus using the recommended PCR protocol due to the lack of the required fluorescent channel (Cy5) in their real-time thermocyclers.

The results of the generic antibody detection exercise were excellent, with 100% correct results in all labs except for one negative sample that one lab reported as doubtful. One limitation of this EQA is the lack of sera with antibodies directed against other flaviviruses, especially considering the high degree of cross-reactions that occur in serological assays [16]. Sera from USUV or JEV infected animals in sufficient amount to prepare an EQA panel are difficult to obtain unless they originate from experimentally infected horses. Unfortunately, we could not have access to this type of samples and, therefore, the panel was restricted to WNV positive and negative sera. As a result, we can only evaluate the capacity of the labs to successfully identify WNV antibodies, but we cannot assess the potential interference of cross-reactivity with other flaviviruses on the performance of the ELISA kits.

More difficulties were observed with the specific detection of IgM antibodies. This was most probably due to the fact that two of the 4 IgM positive sera had low IgM antibody titers with OD values close to the reference threshold. In fact, most of the incorrect results were false negatives in these two sera, while no false positive results were reported. The three labs that used an alternative kit (IDVet IgM capture ELISA) to evaluate the presence of IgM WNV antibodies were only able to detect the sample with the highest IgM titer. These results are in agreement with the data derived from the serology EQA organized by ANSES in 2013, where the INgezim WNV IgM ELISA displayed higher analytical sensitivity than the IDVet IgM capture ELISA for L1 and L2 WNV infected horses [29].

At the end of the exercise, each participant laboratory received an individual report with the analysis of their results, possible reasons for the observed deviations and recommendations to improve their competence.

The unique characteristics of this EQA, where all the participant labs used the same protocols and reagents, enables a comprehensive evaluation of the selected diagnostic methods under “controlled” conditions. In this way, we could verify that the triplex RRT-PCR protocol [15] is a reliable method for accurate detection and differentiation of WNV L1, L2 and USUV in animal samples of different type and origin. This protocol was easily transferred to all the labs that, in most cases, were able to correctly apply the assay and interpret the results. In the current epidemiological context, this method can be a very useful tool for clinical diagnostic and epidemiological surveillance of WNV and USUV. With respect to the pan-flavivirus conventional RT-PCR, more variability of results was observed, especially in samples with low viral loads. Three false positive results were also reported that could be due to the presence of unspecific bands or cross-contamination. However, this broad-range flavivirus assay is a good first-line tool for rapid flavivirus detection and a useful complement to species specific assays. Moreover, this technique allows further genome sequencing to identify the involved virus and perform phylogenetic analysis [35].

In the case of the antibody detection exercise, our results confirm the optimal performance of the Ingenasa competitive ELISA (INgezim West Nile Compac), with high reproducibility values, as all the labs reported concordant results. This commercial kit is widely used for WNV surveillance in birds and horses due to its excellent sensitivity and specificity values [16,29]. The recommended IgM ELISA (INgezim WNV IgM ELISA) also displayed good results and was able to identify positive samples that were not detected with other commercial kits.

This exercise offers a good overview of the WNV and USUV diagnostic capacities of veterinary labs in 17 EU-neighboring countries. Based on the obtained results, most of the participant labs have the necessary infrastructure and expertise to correctly perform the molecular and serological diagnosis of WNV. The training strategy developed during the MediLabSecure project, with two workshops (molecular and serological diagnosis) followed by this EQA, was beneficial in improving the capacities of the labs.

## 4. Materials and Methods

### 4.1. Call for Participation

An invitation letter was sent by the coordinating team of the MediLabSecure animal virology network (INIA-CISA, Madrid, Spain) to the beneficiary veterinary laboratories (*n* = 18). Seventeen laboratories accepted to participate (94.4%). The participation was free of charge and entailed the publication of comparative results in an anonymous manner.

### 4.2. Preparation of EQA Panel

#### 4.2.1. Samples for Virus Genome Detection

For the molecular diagnosis of WNV, each participant received a coded panel of 10 samples, as shown in Table 1.

Four viral strains were used for the preparation of the panel: SP07 strain (WNV L1), isolated from a golden eagle in Spain in 2007 [36]; AUS08 strain (WNV L2), isolated from a goshawk in Austria in 2008 [37], USU11 (Usutu virus) isolated from a blackbird in Italy in 2011 (GenBank number KX816649) [38] and the Nakayama strain (Japanese encephalitis virus-JEV). All the viral stocks were inactivated using ß-propiolactone. Absence of residual infectivity was confirmed after three consecutive passages in Vero cells by absence of cyto-phatic effect and by RRT-PCR analysis.

Several dilutions of inactivated viral stocks were spiked in different matrices (serum, blood, liver, heart or kidney) from healthy non-infected birds and horses to prepare the positive samples. The negative samples consisted of brain and heart homogenates from healthy birds and horses. Nucleic acid extraction was performed from 200 µL of sample using the QIAamp^®^ Cador Pathogen Mini Kit (QIAGEN), following the manufacturer’s instructions. In the final step, RNA was eluted in 50 μL of nuclease-free water. All samples were tested twice with two validated and widely used PCR techniques that we selected as recommended methods: a conventional RT-PCR for pan-flavivirus detection [35] and a RRT-PCR for simultaneous WNV and USUV detection [15].

For the conventional RT-PCR, mix was prepared in a final volume of 25 µL per sample containing 2 µL of RNA template, 0.6 µM of each primer (cFD2 and MAMD), RT-PCR enzyme mix and RT-PCR buffer of the commercial SuperScript^®^ III One-Step RT-PCR System with Platinum^®^ Taq DNA polymerase (Life Technologies, Thermo Fisher Scientific). All reactions were carried out using the following thermal profile: reverse transcription at 55 °C for 30 min, initial PCR activation step at 94 °C for 2 min, followed by 40 cycles of 30 s at 94 °C, 30 s at 55 °C, and 30 s at 68 °C and a final extension step of 5 min at 68 °C. Amplified products were analyzed by 2% agarose gel electrophoresis. Positive samples should give a specific band of the same size as the positive control (252 bp).

The RRT-PCR was performed using the primers, probes and the thermal profile described by del Amo et al. [15]. Samples with Ct > 40 were considered negative.

According to the obtained bands in the conventional RT-PCR and the Ct values in the triplex RRT-PCR, a collection of 10 samples was finally selected (Table 1 and Table 2). The samples were aliquoted (1 mL) and each vial was lyophilized and stored at 4 °C until delivery to the participant laboratories.

Prior to delivery, the lyophilized panel was resuspended in DNAse-free water and was fully analyzed to verify the integrity of the samples and the reproducibility of the results after lyophilization. Triplicates of each lyophilized sample were analyzed by 3 technicians at INIA-CISA using the mentioned techniques. For the RRT-PCR, the reference Ct value was established as the mean of the nine repetitions (Table 2).

Two positive controls were delivered with the panel: (1) a triplex positive extraction control consisting of cell culture medium spiked with a mix of inactivated WNV L1, L2 and USUV strains to obtain, after a 1/10 dilution, an expected Ct value of 32 ± 2 for each virus (this sample was lyophilized and stored at 4 °C until delivery) and (2) a triplex positive reaction control consisting of a mix of WNV L1, L2 and USUV RNAs with an expected Ct value of 32 ± 2 for each virus (this sample was stored at −80 °C until delivery).

#### 4.2.2. Samples for Antibody Detection

For the serological diagnosis of WNV, each participant received a panel of 10 samples (six positive and four negative) (Table 3). The positive samples included sera obtained from naturally infected horses in Southern Spain and one positive reference serum from the EU Reference Laboratory for equine diseases. Four of these samples were IgM positive, obtained from recently infected horses (Table 3). The negative samples consisted of one serum from a non-infected horse and a commercial negative horse serum (Biowhittaker). All samples were inactivated by heating at 56 °C for 45 min. Each sample was aliquoted (130 µL) and stored at −20 °C until delivery.

### 4.3. EQA Details

For the molecular detection, two assays were proposed. First, for generic detection of flaviviruses we recommended the hemi-nested conventional RT-PCR described by Scaramozzino et al. [35] with some modifications as described in the previous section. This RT-PCR targets the highly conserved NS5 region and it is a useful first-line molecular screening test for an unknown flavivirus. However, definitive flavivirus determination requires post-amplification identification techniques (e.g., genome sequencing) or the application of RT-PCR techniques for species-specific detection of flaviviruses. For this reason, the second recommended assay was a triplex RRT-PCR that enables simultaneous detection and differentiation of WNV L1, L2 and USUV [15]. This is based on different sets of primers and fluorogenic probes specific to each virus that are labelled with selective, non-overlapping fluorogen-quencher pairs (FAM for WNV L1, VIC for WNV L2 and Cy5 for USUV). This multiplex RRT-PCR is very sensitive and specific and has been widely validated with experimental and field samples [15,39]. Taking into account the epidemiological situation of the concerned region, where different WNV lineages co-circulate with other related flaviviruses, and especially USUV, this diagnostic approach was considered highly beneficial for surveillance and diagnostic studies.

The labs reported the use of eight different real-time thermocyclers: Rotor-Gene 3000 (5 labs), Applied Biosystems 7500 (5 labs), Applied Biosystems 7300 (2 labs), Aria Mx Agilent (1 lab), Bioer Gene Max (1 lab), Abi QuantStudio (1 lab), StepOnePlus (1 lab) and Stratagene Mx3005 (1 lab).

For serological diagnosis of WNV infection, two commercially available ELISA tests were recommended. The first was the INgezim West Nile Compac ELISA kit (Ingenasa) that allows the detection of anti-E domain III antibodies [40]. It is a multispecies ELISA that only requires 10 µL of sample which is very advantageous for the analysis of sera from small birds. Several studies have proved that this assay is more specific than other commercial ELISAs that, although designed to identify WNV antibodies, also detect cross-reacting antibodies directed against other flaviviruses and especially USUV [16,29]. Moreover, the INgezim West Nile Compac ELISA detects both IgG and IgM antibodies and, based on the results of previous EQAs, it seems that this kit enables more efficient detection of recently-infected animals than other commercial kits [29]. To specifically identify recent (acute) infection in horses, we selected an IgM antibody capture ELISA (MAC-ELISA) from the same company, the Ingezim WNV IgM ELISA. This assay was recommended based on our own comparative studies and on the results of an EQA organized in 2013 where this kit demonstrated the highest analytical sensitivity in horses experimentally infected with WNV L1 and L2 [29].

Detailed standard operating procedures for the recommended assays were distributed and all the reagents and kits were provided to each laboratory, including the mentioned ELISA kits, the extraction kit (QIAamp^®^ Cador Pathogen Mini Kit, QIAGEN), the RT-PCR kit (SuperScript^®^ III One-Step RT-PCR System, Invitrogen) and primers for generic flavivirus detection, the RRT-PCR kit (QuantiTect Probe RT-PCR kit, QIAGEN), and primers and probes for the triplex RRT-PCR. As explained earlier, extraction and reaction positive controls were also delivered to all the labs.

For the molecular panel, specific instructions were provided to reconstitute the lyophilized samples as well as to prepare the positive extraction control. For the serological panel, the laboratories were asked to analyze the panel samples using the recommended kits following manufacturer’s instructions. Additionally, the labs were encouraged to analyze both panels using alternative methods (other protocols that may be established in the labs) and report the results together with those derived from the recommended methods.

The extraction kit, the lyophilized samples, the positive extraction control and the ELISA kits were shipped at room temperature. The panel of sera, the RT-PCR kits, the positive reaction control and the primers and probes were shipped in dry ice. A number code was assigned to each laboratory to ensure a blind analysis of the results.

## Figures and Tables

**Table 1:** Results of the conventional reverse-transcription polymerase chain reaction (RT-PCR) for generic detection of flaviviruses.

**Virus**	**West Nile Virus (WNV) L1**		**WNV L2**		**Usutu Virus (USUV)**	**Japanese Encephalitis Virus (JEV)**	**WNV L1/** **USUV**	**WNV L2/** **USUV**	**--**	**--**	
Strain	SP07		AUS08		USU11	Nakayama	SP07/ USU11	AUS08/USU11	*--*	*--*	
Matrix (species)	Kidney (pheasant)	Liver (pheasant)	Serum (horse)		Blood (horse)	Serum (horse)	Heart (partridge)	Serum (horse)	Brain (horse)	Heart (pheasant)	
Dilution	10^−2^	10^−3^	10^−1^	10^−3^	10^−3^	10^−2^	10^−4^/10^−4^	10^−2^/10^−4^	**--**	**--**	
Sample ID	W10	W1	W2	W5	W6	W9	W3	W8	W4	W7	
Reference value	Weak +	Weak +	Strong +	+	+	+	Weak +	Strong +	-	-	
Laboratory											% of correct results (by lab)
1	NA	NA	NA	NA	NA	NA	NA	NA	NA	NA	--
2	-	-	+	+	+	+	-	+	-	-	70
3	+	-	+	+	+	+	-	+	-	-	80
4	NA	NA	NA	NA	NA	NA	NA	NA	NA	NA	--
5	+	+	+	+	+	+	+	+	-	-	100
6	NA	NA	NA	NA	NA	NA	NA	NA	NA	NA	--
7	+	+	+	+	+	+	+	+	-	-	100
8	+	+	+	+	+	-	-	+	+	+	60
9	+	-	+	-	-	+	-	+	-	-	60
10	-	-	+	+	+	+	-	+	-	-	70
11	+	+	+	+	+	+	+	+	-	-	100
12	+	+	+	+	+	+	+	+	-	-	100
13	-	+	+	-	+	-	-	+	-	+	50
14	+	+	+	+	+	+	+	+	-	-	100
15	+	+	+	+	+	+	+	+	-	-	100
16	-	-	+	+	+	-	-	+	-	-	60
17	NA	NA	NA	NA	NA	NA	NA	NA	NA	NA	--
% of correct results (by sample)	69.2	61.5	100	84.6	92.3	76.9	46.2	100	92.3	84.6	

Blue: false negative results; Red: false positive results; NA: not analyzed.

**Table 2:** Results of the triplex RRT-PCR for detection and differentiation of WNV L1, L2 and USUV.

**Virus**		**WNV L1**		**WNV L2**		**USUV**	**JEV**	**WNV L1/USUV**	**WNV L2/USUV**	**--**	**--**		
Strain		SP07		AUS08		USU11	Nakayama	SP07/ USU11	AUS08/ USU11	*--*	*--*		
Matrix (species)		Kidney (pheasant)	Liver (pheasant)	Serum (horse)		Blood (horse)	Serum (horse)	Heart (partridge)	Serum (horse)	Brain (horse)	Heart (pheasant)		
Dilution		10^−2^	10^−3^	10^−1^	10^−3^	10^−3^	10^−2^	10^−4^/10^−4^	10^−2^/10^−4^	--	--		
Sample ID		W10	W1	W2	W5	W6	W9	W3	W8	W4	W7		
Reference Ct value	WNV L1 (FAM)	30.68 ± 0.88	33.60 ± 0.98	No Ct	No Ct	No Ct	No Ct	32.01 ± 0.41	No Ct	No Ct	No Ct		
	WNV L2 (VIC)	No Ct	No Ct	27.13 ± 0.57	34.28 ± 0.13	No Ct	No Ct	No Ct	31.34 ± 0.25	No Ct	No Ct		
	USUV (Cy5)	No Ct	No Ct	No Ct	No Ct	29.24 ± 0.46	No Ct	32.1 ± 0.58	33.23 ± 0.31	No Ct	No Ct		
Lab												% of correct results for each virus (by lab)	% of correct overall results (by lab) ^†^
1	WNV L1 (FAM)	No Ct	No Ct	32.79	No Ct	No Ct	No Ct	No Ct	No Ct	31.98	No Ct	60	10
	WNV L2 (VIC)	No Ct	No Ct	No Ct	No Ct	34.59	30	24.8	No Ct	No Ct	No Ct	40	
	USUV (Cy5)	No Ct	No Ct	No Ct	No Ct	No Ct	38	No Ct	No Ct	33.53	36.2	50	
2	WNV L1 (FAM)	27.75	33.32	No Ct	No Ct	No Ct	No Ct	32.4	No Ct	No Ct	No Ct	100	100
	WNV L2 (VIC)	No Ct	No Ct	37.04	32.87	No Ct	No Ct	No Ct	30.71	No Ct	No Ct	100	
	USUV (Cy5)	No Ct	No Ct	No Ct	No Ct	37.57	No Ct	34.1	36.19	No Ct	No Ct	100	
3	WNV L1 (FAM)	30.15	31.71	No Ct	No Ct	No Ct	No Ct	30.31	No Ct	No Ct	No Ct	100	100
	WNV L2 (VIC)	No Ct	No Ct	21.66	26.66	No Ct	No Ct	No Ct	23.12	No Ct	No Ct	100	
	USUV (Cy5)	No Ct	No Ct	No Ct	No Ct	28.89	No Ct	28.58	30.24	No Ct	No Ct	100	
4	WNV L1 (FAM)	28.28	31.07	No Ct	No Ct	No Ct	No Ct	30.44	No Ct	No Ct	No Ct	100	100
	WNV L2 (VIC)	No Ct	No Ct	24.53	32.55	No Ct	No Ct	No Ct	28.01	No Ct	No Ct	100	
	USUV (Cy5)	No Ct	No Ct	No Ct	No Ct	24.8	No Ct	27.45	28.02	No Ct	No Ct	100	
5	WNV L1 (FAM)	29.44	32.74	No Ct	No Ct	No Ct	No Ct	30.52	No Ct	No Ct	No Ct	100	100
	WNV L2 (VIC)	No Ct	No Ct	24.67	32.65	No Ct	No Ct	No Ct	28.06	No Ct	No Ct	100	
	USUV (Cy5)	No Ct	No Ct	No Ct	No Ct	31.89	No Ct	36.92	33.74	No Ct	No Ct	100	
6	WNV L1 (FAM)	31.6	36.8	No Ct	No Ct	No Ct	No Ct	34.5	No Ct	No Ct	No Ct	100	70
	WNV L2 (VIC)	No Ct	No Ct	30.2	38.7	No Ct	No Ct	No Ct	33.9	No Ct	No Ct	100	
	USUV (Cy5)	No Ct	No Ct	No Ct	No Ct	No Ct	No Ct	No Ct	No Ct	No Ct	No Ct	70	
7	WNV L1 (FAM)	31.84	31.88	No Ct	No Ct	No Ct	No Ct	32.3	No Ct	No Ct	No Ct	100	70
	WNV L2 (VIC)	No Ct	No Ct	25.54	32.78	No Ct	No Ct	No Ct	29.87	No Ct	No Ct	100	
	USUV (Cy5)	No Ct	No Ct	No Ct	No Ct	No Ct	No Ct	No Ct	No Ct	No Ct	No Ct	70	
8	WNV L1 (FAM)	31.2	No Ct	No Ct	No Ct	No Ct	No Ct	33.46	No Ct	No Ct	No Ct	90	90
	WNV L2 (VIC)	No Ct	No Ct	31	35.5	No Ct	No Ct	No Ct	32.5	No Ct	No Ct	100	
	USUV (Cy5)	NA	NA	NA	NA	NA	NA	NA	NA	NA	NA	-	
9	WNV L1 (FAM)	29	32	No Ct	No Ct	No Ct	No Ct	29	No Ct	No Ct	No Ct	100	100
	WNV L2 (VIC)	No Ct	No Ct	27	37	No Ct	No Ct	No Ct	32	No Ct	No Ct	100	
	USUV (Cy5)	No Ct	No Ct	No Ct	No Ct	28	No Ct	32	34	No Ct	No Ct	100	
10	WNV L1 (FAM)	28.19	30.76	No Ct	No Ct	No Ct	No Ct	29.59	No Ct	No Ct	No Ct	100	100
	WNV L2 (VIC)	No Ct	No Ct	22.11	29.8	No Ct	No Ct	No Ct	26.78	No Ct	No Ct	100	
	USUV (Cy5)	No Ct	No Ct	No Ct	No Ct	28.39	No Ct	30.38	30.97	No Ct	No Ct	100	
11	WNV L1 (FAM)	29.03	31.57	No Ct	No Ct	No Ct	No Ct	30.96	No Ct	No Ct	No Ct	100	100
	WNV L2 (VIC)	No Ct	No Ct	25.46	32.65	No Ct	No Ct	No Ct	28.75	No Ct	No Ct	100	
	USUV (Cy5)	No Ct	No Ct	No Ct	No Ct	28.81	No Ct	29.46	30.95	No Ct	No Ct	100	
12	WNV L1 (FAM)	32.32	34.5	No Ct	No Ct	No Ct	No Ct	32.09	No Ct	No Ct	No Ct	100	100
	WNV L2 (VIC)	No Ct	No Ct	29.39	35.69	No Ct	No Ct	No Ct	32.11	No Ct	No Ct	100	
	USUV (Cy5)	NA	NA	NA	NA	NA	NA	NA	NA	NA	NA	-	
13	WNV L1 (FAM)	32.38	34.03	No Ct	No Ct	No Ct	No Ct	33.69	No Ct	No Ct	No Ct	100	100
	WNV L2 (VIC)	No Ct	No Ct	27.8	36.3	No Ct	No Ct	No Ct	31.45	No Ct	No Ct	100	
	USUV (Cy5)	No Ct	No Ct	No Ct	No Ct	36	No Ct	36.7	37.7	No Ct	No Ct	100	
14	WNV L1 (FAM)	28	32	No Ct	No Ct	No Ct	No Ct	31.6	No Ct	No Ct	No Ct	100	100
	WNV L2 (VIC)	No Ct	No Ct	26.4	30.6	No Ct	No Ct	No Ct	26.4	No Ct	No Ct	100	
	USUV (Cy5)	No Ct	No Ct	No Ct	No Ct	30.8	No Ct	32.5	32.8	No Ct	No Ct	100	
15	WNV L1 (FAM)	31.66	32.86	No Ct	No Ct	No Ct	No Ct	34.11	No Ct	No Ct	No Ct	100	100
	WNV L2 (VIC)	No Ct	No Ct	23.19	32.84	No Ct	No Ct	No Ct	27.41	No Ct	No Ct	100	
	USUV (Cy5)	No Ct	No Ct	No Ct	No Ct	29.45	No Ct	35.73	29.6	No Ct	No Ct	100	
16	WNV L1 (FAM)	32	31.9	No Ct	No Ct	No Ct	No Ct	31.2	No Ct	No Ct	No Ct	100	100
	WNV L2 (VIC)	No Ct	No Ct	24	33.5	No Ct	No Ct	No Ct	32.8	No Ct	No Ct	100	
	USUV (Cy5)	No Ct	No Ct	No Ct	No Ct	27.8	No Ct	32.6	28.2	No Ct	No Ct	100	
17	WNV L1 (FAM)	34	30	No Ct	No Ct	No Ct	No Ct	32	No Ct	No Ct	No Ct	100	80
	WNV L2 (VIC)	No Ct	No Ct	28	34	No Ct	No Ct	No Ct	37	32	No Ct	90	
	USUV (Cy5)	No Ct	No Ct	No Ct	No Ct	31	No Ct	28	No Ct	No Ct	No Ct	90	
% of correct results (by sample) ^‡^	94.1	88.2	94.1	94.1	82.3	94.1	82.3	76.4	94.1	94.1	94.1		

Blue: false negative results; Red: false positive results; NA: not analyzed due to lack of appropriate fluorophore (Cy5) ^†^ Percentage of samples where all the viruses have been correctly identified; ^‡^ Percentage of 100% correct results by sample (all the viruses of each sample have been correctly identified).

**Table 3:** Results of the external quality assessment (EQA) for WNV IgM antibody detection using the recommended method (INgezim West Nile IgM ELISA).

Serum Sample	Horse (past infection)		Horse (recent infection)				Non-infected horse		Negative commercial horse serum		
	W3	W6	W2	W8	W10	W4	W1	W9	W5	W7	
Reference OD values	0.11 ± 0.05	0.25 ± 0.21	0.34 ± 0.04	0.44 ± 0.09	0.48 ± 0.12	1.82 ± 0.21	0.07 ± 0.03	0.12 ± 0.11	0.10± 0.11	0.05 ± 0.03	
Reference qualitative result	-	-	D/+	+	+	+	-	-	-	-	
Laboratory											% of correct results (by lab)
1	-	-	+	-	+	+	-	-	-	-	90
2	-	-	D	+	+	+	-	-	-	-	100
3	-	-	+	+	+	+	-	-	-	-	100
4	-	-	+	+	+	-	-	-	-	-	90
5	-	-	+	+	+	+	-	-	-	-	100
6	-	-	-	-	D	+	-	-	-	-	70
7	-	-	+	+	+	+	-	-	-	-	100
8	NA	NA	NA	NA	NA	NA	NA	NA	NA	NA	--
9	-	-	+	+	+	+	-	-	-	-	100
10	-	-	D	-	+	+	-	-	-	-	90
11	-	-	+	+	+	+	-	-	-	-	100
12	-	-	+	+	+	+	-	-	-	-	100
13	-	-	+	+	+	+	-	-	-	-	100
14	-	-	D	D	+	+	-	-	-	-	90
15	-	D	D	+	+	+	-	-	-	-	90
16	-	-	D	+	+	+	-	-	-	-	100
17	-	-	-	D	+	+	-	-	-	-	80
% of correct results (by sample)	100	93.7	87.5	68.7	93.7	93.7	100	90	100	100	

Grey: incorrect result; NA: not analyzed; D: doubtful.
